# The lung cancer exercise training study: a randomized trial of aerobic training, resistance training, or both in postsurgical lung cancer patients: rationale and design

**DOI:** 10.1186/1471-2407-10-155

**Published:** 2010-04-21

**Authors:** Lee W Jones, Neil D Eves, William E Kraus, Anil Potti, Jeffrey Crawford, James A Blumenthal, Bercedis L Peterson, Pamela S Douglas

**Affiliations:** 1 Duke University Medical Center, Durham, NC, USA; 2 University of Calgary, Calgary, Alberta, Canada

## Abstract

**Background:**

The Lung Cancer Exercise Training Study (LUNGEVITY) is a randomized trial to investigate the efficacy of different types of exercise training on cardiorespiratory fitness (VO_2peak_), patient-reported outcomes, and the organ components that govern VO_2peak _in post-operative non-small cell lung cancer (NSCLC) patients.

**Methods/Design:**

Using a single-center, randomized design, 160 subjects (40 patients/study arm) with histologically confirmed stage I-IIIA NSCLC following curative-intent complete surgical resection at Duke University Medical Center (DUMC) will be potentially eligible for this trial. Following baseline assessments, eligible participants will be randomly assigned to one of four conditions: (1) aerobic training alone, (2) resistance training alone, (3) the combination of aerobic and resistance training, or (4) attention-control (progressive stretching). The ultimate goal for all exercise training groups will be 3 supervised exercise sessions per week an intensity above 70% of the individually determined VO_2peak _for aerobic training and an intensity between 60 and 80% of one-repetition maximum for resistance training, for 30-45 minutes/session. Progressive stretching will be matched to the exercise groups in terms of program length (i.e., 16 weeks), social interaction (participants will receive one-on-one instruction), and duration (30-45 mins/session). The primary study endpoint is VO_2peak_. Secondary endpoints include: patient-reported outcomes (PROs) (e.g., quality of life, fatigue, depression, etc.) and organ components of the oxygen cascade (i.e., pulmonary function, cardiac function, skeletal muscle function). All endpoints will be assessed at baseline and postintervention (16 weeks). Substudies will include genetic studies regarding individual responses to an exercise stimulus, theoretical determinants of exercise adherence, examination of the psychological mediators of the exercise - PRO relationship, and exercise-induced changes in gene expression.

**Discussion:**

VO_2peak _is becoming increasingly recognized as an outcome of major importance in NSCLC. LUNGEVITY will identify the optimal form of exercise training for NSCLC survivors as well as provide insight into the physiological mechanisms underlying this effect. Overall, this study will contribute to the establishment of clinical exercise therapy rehabilitation guidelines for patients across the entire NSCLC continuum.

**Trial Registration:**

NCT00018255

## Background

Improvements in surgical techniques together with more effective adjuvant chemotherapeutic regimens has led to significant survival benefit for individuals with non-small cell lung cancer (NSCLC). Approximately 26,000 individuals per year in the United States will survive more than 5 years after initial diagnosis of operable disease. With improving prognosis, acute and long-term disease - and treatment-related morbidity (symptom control) and mortality are now recognized as issues of major clinical importance in the multidisciplinary management of operable NSCLC [1-6]. A parameter of central importance that may mediate acute and late-occurring disease and treatment-related toxicity in lung cancer survivorship is cardiorespiratory fitness. Cardiorespiratory fitness, as measured by an objective exercise tolerance test, reflects the integrative capacity of components in the oxygen (O_2_) cascade to supply adequate O_2 _for adenosine triphosphate (ATP) resynthesis. Peak oxygen consumption (VO_2peak_) provides the gold standard (direct) assessment of cardiorespiratory fitness. Direct or estimated measurement of cardiorespiratory fitness is a well-established independent predictor of mortality in a broad range of non-cancer, adult populations [7,8].

Not surprisingly, operable NSCLC patients have significant and marked reductions in VO_2peak_. Postsurgical NSCLC patients are subject to the effects of normal ageing, age-related and/or disease-related comorbid conditions, and deconditioning that adversely impact components of the O_2 _cascade. However, these 'normal' consequences are profoundly accelerated by disease pathophysiology and the use of conventional adjuvant therapy to create a 'perfect deconditioning storm', reducing either the body's ability to deliver and/or utilize O_2 _and substrate leading to poor VO_2peak_[9]. Such effects have important implications across the entire NSCLC continuum[10].

First, preoperative VO_2peak _is a well-established risk stratification tool to determine perioperaitve and postoperative complication risk [11-14]. Second, following resection, VO_2peak _as well as self-reported exercise behavior (a major determinant of VO_2peak_), are strong predictors of patient-reported outcomes (PROs) such as overall QOL, fatigue, and other QOL domains[15]. Finally, our group found that pre-operative VO_2peak _is a strong independent predictor of overall survival in NSCLC surgical candidates even when controlling for performance status, gender, and age[16]. In totality, these data provide strong evidence that VO_2peak _is an attractive modifiable therapeutic target to improve surgical risk and/or recovery, symptom control and possibly, disease outcome in NSCLC.

Chronic, repeated aerobic training (i.e., continuous activity involving large muscle groups) is widely established as the most effective method to improve VO_2peak _in healthy humans although a paucity of studies have investigated the role of exercise in NSCLC[10]. Given the preliminary nature of this field, we recently completed two uncontrolled pilot studies investigating the feasibility and preliminary efficacy of supervised aerobic training in the pre-operative and post-operative setting in NSCLC. Results of these pilot studies provided 'proof of principle' that aerobic training is a safe and feasible intervention for NSCLC patients, however, the improvements in VO_2peak _were modest (<15%), particularly in the post-operative setting (~10%) despite good exercise adherence rates (≥70% of planned sessions) [17,18].

The reasons for the relatively modest improvement in VO_2peak _in NSCLC relative to other clinical populations (i.e., ~15%-20% improvement in VO_2peak _following traditional aerobic training recommendations) remain to be elucidated. An obvious potential explanation is a ventilatory limitation or inadequate gas exchange following removal of a substantial portion of lung parenchyma. However, several elegant studies have demonstrated that VO_2peak _is not limited by ventilation or diffusion capacity [19-22] suggesting that exercise-induced adaptations (or lack thereof) in the other organ components of the O_2 _cascade are responsible. VO_2peak _in NSCLC patients is likely principally governed by poor cardiovascular O_2 _delivery and oxidative capacity *as well as *unfavorable fiber type distribution and muscle atrophy/weakness similar to the limitations to exercise described in patients with chronic obstructive pulmonary disease (COPD). Major contributors to skeletal muscle dysfunction in NSCLC likely include direct skeletal myopathy (from the use of oral corticosteroids), deconditioning (from physical inactivity), and high levels of systemic inflammation (from underlying disease and therapy)[23].

**Figure 1:**
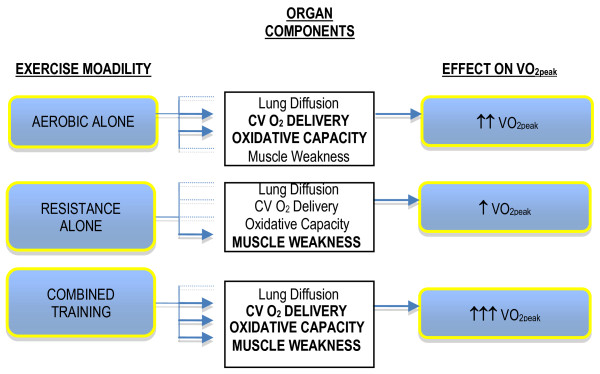
**Hypothesized effects of aerobic training alone, resistance training alone, and aerobic plus resistance training on the components of the oxygen cascade and resultant impact on exercise tolerance (VO_2peak_)**.

Importantly, aerobic training will cause favorable adaptations in most O_2 _transport components but will not reverse skeletal muscle atrophy/weakness and will only partially reverse a more glycolytic fiber type distribution. Thus, aerobic training alone may be insufficient to ameliorate skeletal muscle dysfunction likely manifest in NSCLC. Standard resistance training (i.e., activity involving the acute exertion of force) performed according to standard guidelines (i.e., 2-5 times/week, 50%-80% of 1 repetition maximum for 12-24 weeks) is unequivocally acknowledged as the most effective method to improve skeletal muscle function in human subjects [24-28]. Moreover, in severely deconditioned adults, resistance training causes improvements in VO_2peak _[29-34] although the mechanisms underlying this effect are not clearly understood. In theory, while aerobic and resistance training might independently improve VO_2peak _in NSCLC, such improvements are likely to be modest (~10%). Instead, the combination of aerobic and resistance training may be the most effective form of exercise training to optimally augment VO_2peak_. The complementary physiologic adaptations from the combination approach will result in higher cardiovascular O_2 _delivery, skeletal muscle oxidative phosphoryation, muscle strength and optimal fiber type composition leading to higher muscle endurance, reduced fatiguability, a higher threshold to the metabolic waste products of exercise, and reduced ventilatory requirements during exercise. This approach is hypothesized to maximize physiologic adaptations in the principal factors underlying poor VO_2peak _in postsurgical NSCLC patients more effectively than either exercise modality alone (Figure 1).

Against this background, we designed the Lung Cancer Exercise Training Study (LUNGEVITY), a randomized trial to investigate the efficacy of different types of exercise training in post-operative NSCLC patients. The fundamental rationale for this single-center trial is to identify the optimal type of exercise training to improve VO_2peak _in postoperative NSCLC patients and understand the physiologic mechanisms underlying this effect. The specific aims are: (1) to compare the effects of aerobic training alone, resistance training alone, or both, relative to attention-control, on VO_2peak_, (2) determine the effects on the mechanisms that govern VO_2peak _(measurements of the heart-lung-skeletal muscle axis), and (3) to compare the effects on PROs.

## Methods/Design

### Participants and Setting

In LUNGEVITY, we will recruit and randomize 160 subjects (40 patients/study arm) with histologically confirmed stage I-IIIA NSCLC following curative-intent complete surgical resection at Duke University Medical Center (DUMC). The DUMC institutional review board approved the study and written informed consent will be obtained from all participants prior to initiation of any study procedures. Additional inclusion and exclusion criteria are described in Table 1.

**Table 1:** Subject Eligibility Criteria

Inclusion Criteria	
At least 21 years old.	
An interval of at least 6 months following surgical resection.	VO_2peak _has been shown to (spontaneously) recover, to a limited degree, immediately following (~3 months) and stabilize at ~6 months post pulmonary resection. Thus, to accurately determine the effects of exercise training on VO_2peak _in this setting, we felt it was critically important to initiate study procedures (i.e., recruit and randomize patients) once changes in VO_2peak _have stabilized to minimize the effects of natural postsurgical recovery on improvements in VO_2peak_,
An interval of no longer than 36 months post-resection	
Karnofsky performance status of at least 70% at study entry	
Estimated life expectancy of ≥6 months	
Ability to read and understand English	
Primary attending oncologist approval	
Sedentary	Patients not performing regular exercise. Regular exercise is defined as ≥5 days a week, ≥30 minutes each session, at a moderate or vigorous intensity for the past month).
Willingness to be randomized	
Signed informed consent prior to initiation of study-related procedures	
Reside within driving distance of DUMC, as necessitated by the clinic-based assessments and supervised exercise training interventions	
	
Exclusion Criteria	
Presence of a concurrent, actively treated other malignancy or history of other malignancy treated within the past 3 years (other than non-melanoma skin cancer)	
Presence of metastatic disease	
Scheduled to receive any form of adjuvant cancer therapy	
Contraindications to maximal exercise testing as recommended by the American Thoracic Society and exercise testing guidelines for cancer patients	

### Procedures

**Figure 2:**
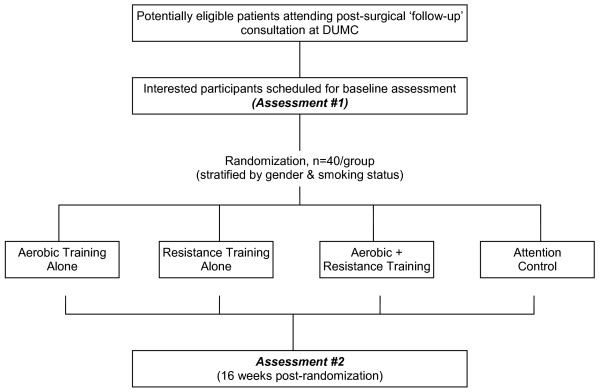
**Study Flow**.

The study will be conducted in accordance with the CONSORT (Consolidated Standards of Reporting Trials) statement for non-pharmacologic interventions[35]. The study flow is presented in Figure 2. Using a 4-arm, randomized design, potential subjects will be identified and screened for eligibility by the study research coordinators via medical record review of NSCLC patients scheduled to attend a 'follow-up' consultation at DUMC as per standard of care. Following the consultation and primary attending oncologist approval, potential eligible subjects will be provided with a thorough review of the study by the study coordinators and asked if they are willing to participate. Interested participants will be provided with the study consent and baseline study questionnaire. Two to five days following the consultation, interested participants will be contacted by telephone by the study coordinators to answer any questions and to schedule the baseline assessment visit. In addition, to broaden recruitment efforts, subjects previously diagnosed with stage I-IIIA NSCLC within 36 months of diagnosis will be identified through the DUMC tumor registry. Permission to contact potential subjects will be obtained from their attending oncologist and letters of study invitation describing the study co-signed by the principal investigator and attending oncologist will be mailed to all potential subjects. Interested participants will be able to call a toll-free telephone number to obtain more information about study participation.

At the baseline study visit, all participants will complete the following assessments in order of presentation: (1) fasting blood draw, (2) pulmonary function test (3) cardiopulmonary exercise test, and (4) echocardiogram at rest and exercise. On the following day, patients will perform the following assessments: (1) one repetition maximum lower extremity strength test, and (2) tissue biopsy of the vastus lateralis. Participants will be asked to adhere to a water-only fast for 8 hours prior to testing on both days. All baseline assessments will be repeated at the end of the intervention (i.e., 16 weeks). To maximize internal validity, study endpoint assessments will be conducted by the same personnel, with the same equipment, in the same order (timing) at baseline and follow-up timepoints.

### Group Allocation (Randomization)

Following the successful completion of baseline assessments, participants will be randomly allocated, on an individual basis, to one of the three exercise interventions (i.e., aerobic training, resistance training, the combination of aerobic and resistance training) or an attention-control group. Randomly allocated participants will remain in the same group for the entire duration of the intervention (i.e., no cross-over). To ensure randomized groups are similar at baseline, patient randomization will be stratified based on gender (men vs. female) and smoking status (current vs. non-smoking). For each gender-smoking status participant subgroup, a computer-generated list of random treatment assignments will be created by Dr. Peterson (trial biostatistician) in sequentially numbered sealed envelopes. The random allocation sequences will be concealed from all study personnel (except Dr. Peterson). A permuted block design with allocation weight of 1:1:1:1 will be used to generate the treatment assignments.

### Blinding/Masking

Blinding study participants to treatment allocation in clinical trials of exercise training is not possible since participants are aware whether they are exercising or not. Also, it is not possible to blind study personnel monitoring exercise training sessions to the participant group assignment since study personnel are aware whether a participant is performing aerobic or resistance training. Nevertheless, all study personnel conducting the study assessments at baseline and postintervention will be blinded to treatment assignment for the duration of the study. Only the trial statistician and the data monitoring committee will have access to unblinded data, but none will have any contact with study participants or study personnel.

### Exercise Training Protocols (General Considerations)

The ultimate goal for all exercise training groups will be 3 supervised exercise sessions per week an intensity above 70% of the individually determined VO_2peak _for aerobic training and an intensity between 60 and 80% of one-repetition maximum for resistance training, for 30-90 minutes/session. All the exercise interventions are designed such that participants begin exercising at a low intensity (~50%-60% VO_2peak_) that is subsequently increased to more moderate to vigorous intensity (~70%-80% VO_2peak_) when appropriate. All interventions will be individually tailored to each patient following the principles of aerobic or resistance training prescription guidelines for adults as recommended by the American College of Sports Medicine (ACSM)[24]. All exercise sessions will also be performed in a supervised setting with one-on-one supervision by an ACSM-certified exercise physiologist. Supervised exercise training sessions are critical to ensure a robust test of exercise training efficacy and safety in this setting.

All exercise sessions will include a 5-min warm-up and 5-min cool down on either a treadmill or cycle ergometer at the beginning and end of each training session, respectively. Heart rate and O_2 _saturation will be monitored continuously throughout exercise while blood pressure will be assessed prior, during, and immediately following each exercise session. To monitor any exercise performed outside of the supervised sessions, participants will be provided with a heart rate monitor as well as a study exercise log-book to record all sessions.

#### Study Arm 1: Aerobic Training Alone

Aerobic training will be prescribed according to ASCM guidelines and aimed at increasing VO_2peak_. The ultimate goal for aerobic training alone is 3 cycle ergometry sessions/week at an intensity above 70% of VO_2peak _for 30-60 minutes/session. Cycle ergometry was chosen as the mode of aerobic training because exercise prescriptions can be more accurately prescribed and monitored on cycle ergometers than treadmill walking. Also, treadmill walking requires considerable balance; lung cancer patients are typically older, have balance issues, and typically have limited experience with treadmill exercise. Specific details of the aerobic training prescription are provided in Table 2.

**Table 2:** Aerobic Training Alone Program

		Supervised Aerobic Training		
	
Training Phase	Week	Weekly Sessions	Duration (min/session)	Intensity (% of VO_2peak_)
Introductory	0-4	2-3	15-25	50-60
Intermediate	5-8	3	20-30	60-70
Maintenance	9-16	3	25-45	≥70

*Weeks intervals shown are goals and may vary for individual patients; All exercise sessions must be performed in a supervised setting.

#### Study Arm 2: Resistance Training Alone

Resistance training will be prescribed according the ASCM guidelines and aimed at increasing VO_2peak_. The ultimate goal for resistance training alone is 3 resistance training sessions/week at an intensity above 60% to 80% of one-repetition maximum (1-RM) for 30-60 minutes/session (Table 3). Resistance training will be performed on stationary weight machines (i.e., no free weights). Patients will be progressively trained to perform three sets of 8 to 12 repetitions of 8 resistance exercise alternating between lower and upper body muscle groups.

**Table 3:** Resistance Training Alone Program

		Supervised Resistance Training			
	
Training Phase	Week	Weekly Sessions	Sets, n	Repetitions, n	Weight, maximum repetitions
Introductory	0-4	2-3	1-2	8-12	12
Intermediate	5-8	3	2-3	8-12	12
Maintenance	9-16	3	3	8-10	10

*Weeks intervals shown are goals and may vary for individual patients; All exercise sessions must be performed in a supervised setting.

#### Study Arm 3: Combined Aerobic and Resistance Training

Combined aerobic and resistance training will be prescribed according the ASCM guidelines and aimed at increasing VO_2peak_. The ultimate goal will be three combined exercise sessions per week at an intensity above 60% VO_2peak _and above 60% 1-RM for aerobic and resistance training, respectively for 30-60 minutes/session. The general design of the combined intervention is provided in Table 4 and follows similar intensities to the single modality training. In this arm, the duration of aerobic training and resistance training will not be added together but rather the exercise prescription is designed to exploit the complementary properties of aerobic and resistance training to optimally improve VO_2peak_. The prescription will be balanced to ensure that on days when aerobic training is prescribed at a high-intensity, resistance training (on the same day) will be conducted at a lower (easy) intensity and vice versa. This approach will optimize the desired adaptations without causing excessive fatigue and will help avoid potential interference effects between aerobic and resistance training.

**Table 4:** Combined Aerobic and Resistance Training Program

		Aerobic Training			Resistance Training			
	
Training Phase	Wk*	Weekly Sessions	Duration (min/session)	Intensity of VO_2peak_	Duration (min/session)	Sets, n	Repetitions, n	Weight, max repetitions
Introductory	0-4	2	15-25	50-60	15-20	1	8-12	12
Intermediate	5-9	3	20-30	>70	~25	2	8-12	12
Maintenance	10-24	3	30-45	>70	~30	2	8-10	10

*Week intervals shown are goals and may vary for individual patients. All exercise sessions must be performed in a supervised setting.

### Attention Control Group (Study Arm 4)

Subjects assigned to the attention-control group will perform supervised progressive stretching matched to the exercise interventions in terms of program length (i.e., 16 weeks), social interaction (participants will receive one-on-one instruction), and duration (30-45 mins/session). The progressive stretching program will be prescribed according the ASCM guidelines for older adults and aimed at increasing whole-body flexibility. The ultimate goal for the progressive stretching program is 3 stretching sessions/week for 30-60 minutes/session for 16 weeks (Table 5). Stretching will be performed supine on stretching mats (i.e., no machines). Patients will be progressively trained to perform 8 stretching exercises alternating between lower and upper body muscle groups/joints.

**Table 5:** Attention Control Program

		Supervised Aerobic Training		
	
Training Phase	Week	Weekly Sessions	Duration (min/session)	Duration of each Stretch (mins)
Introductory	0-4	2-3	15-25	1-2
Intermediate	5-8	3	20-30	2-5
Maintenance	9-16	3	25-45	≥5

*Weeks intervals shown are goals and may vary for individual patients; All exercise sessions must be performed in a supervised setting.

### Adherence Considerations

To maximize adherence, several strategies will be employed including individualized attention at the intervention sessions, telephone calls following missed sessions, individuals meetings to outline goals and providing feedback on study progress. In addition, participants will be asked to perform 3 supervised exercise/stretching sessions per week over a 7-day period, and will be allowed to make-up missed sessions within the 16-week study period. Also, participants will be allowed to schedule supervised exercise training sessions at anytime from 7 am to 7 pm. This degree of scheduling flexibility allows participants to perform exercise training sessions at a convenient time and work around other competing demands such as medical appointments, work, and family commitments. Finally, the study team, consisting of the PI, the exercise physiologist and study coordinator, will also meet on a weekly basis to review each participant's adherence with weekly program goals.

### Study Endpoints and Assessments

Table 6 outlines the study assessment schedule while a brief description of study endpoints and endpoint assessments including sub-studies is provided in Table 7.

**Table 6:** Study Assessment Schedule

			Baseline			Postintervention (16 weeks)	
Assessment	Screening	Day 0	Day 1	Day 2	Day 3-5	Day 112	Day 113
Chart Review	x						
Patient Approached	x						
Informed Consent	x						
Day 1 Testing							
Blood draw		x				x	
Body Composition		x				x	
Pulmonary Function		x				x	
CPET		x				x	
Echocardiogram		x				x	
Day 2 Testing							
Strength testing			x				x
Muscle biopsy			x				x
Day 3 Testing							
Repeat CPET				x			
Randomization				x			
Intervention initiation					x		

**Table 7:** Study Measurements and Sub-Studies

Physical measurements and tests
Height, weight, and body mass index
Body composition
Resting and exercise heart rate
Resting and exercise blood pressure
Resting and exercise 12-lead ECG
Peak and submaximal oxygen consumption
Ventilatory threshold

Mechanistic physiological measurements
Resting and exercise echocardiogram
Skeletal muscle function (fiber type distribution, oxidative capacity, and muscular strength)
Pulmonary function
Hemoglobin concentration
Resting and exercise oxygen saturation
Each intervention (exercise or attention control) session: heart rate, blood pressure, oxygen saturation, RPE

Patient-reported outcomes (questionnaire-based)
Medications
Quality of life
Fatigue
Dyspnea
Depression
Adverse events (Common Terminology Criteria for Adverse Events; CTCAE v.4.0)

Substudies
Genetic studies to examine differences to exercise stimulus
Predictors of exercise adherence
Pilot studies for exercise-induced changes in peripheral gene expression

#### Primary Endpoint

*VO*_2peak _will be evaluated using a physician-supervised incremental cycle ergometer test with 12-lead ECG monitoring (Mac^® ^5000, GE Healthcare) will be performed by ACSM-certified exercise physiologists blinded to the patient's randomization group. Expired gases will be analyzed continuously by a metabolic measurement system (ParvoMedics TrueMax, Sandy, UT). Subjects will begin pedaling at 20 Watts for one minute and is increased 5 to 20 Watts every minute until exhaustion or a symptom-limited VO_2peak _is achieved. This protocol has been previously demonstrated to be appropriate for measuring VO_2peak _in our prior studies among NSCLC patients[18,36-38]. Exercise will be terminated if any ECG abnormalities are observed.

### Secondary Endpoints

**Patient Reported Outcomes **will include QOL, fatigue, dyspnea, and depression. *QOL *will be assessed using the Functional Assessment of Cancer Therapy - Lung (FACT-L) scale developed for the assessment of patient symptoms and QOL in lung cancer patients [39]. The FACT-L contains four subscales for physical (7-items), functional (7-items), emotional (6-items), social/family (7-items) well-being plus a lung cancer specific subscale (15-items) which will be summed to obtain the FACT-L score (all 42 items). *Fatigue *will be assessed using the 13-item FACT-fatigue scale for the assessment of fatigue in cancer patients[40]. *Dyspnea *will be assessed using the Cancer Dyspnea Scale (CDS)[41]. The CDS is a 12-item scale comprised of three factors (sense of sense/sense of anxiety/sense of discomfort). This instrument has been demonstrated to be a brief, valid, and feasible scale for assessing cancer-related dyspnea among inoperable NSCLC patients[42,43]. Finally, *depression *will be assessed using the Center for Epidemiologic Studies Depression scale (CES-D)[44]. We have found these instruments to be reliable, valid, responsive, brief, and easy to administer in our on-going study and prior reports in lung cancer patients.

**Physiologic Mechanisms of VO_2peak _**will include pulmonary function, cardiovascular O_2 _delivery, and skeletal muscle function. *Pulmonary Function *will be determined using standard spirometry to assess FEV_1_. All measures will be performed in a sitting position according to the American Thoracic Society guidelines [45]. *Cardiovascular O*_2_*Delivery *will be comprised of: *(1) Cardiac Output*: Left ventricular volumes will be performed with a commercially available ultrasound system (GE Vivid 7 or Philips i33). Apical two- and four- chamber views will be assessed at rest and exercise to determine left ventricular end-diastolic volume and end-systolic volume by modified Simpson's rule [46,47]. Stroke volume will be calculated as end-diastolic volume minus end-systolic volume. Cardiac output will be calculated by stroke volume multiplied by heart rate, (2) *Hemoglobin (Hb) Concentration *(O_2 _carrying capacity of blood) will be assessed via a venous blood draw according to standard guidelines. Normal range for hemoglobin is ~13 - 18 grams per deciliter for men and 12 - 16 for women. Mean cell Hb (amount of Hb/red blood cell) and platelet count (number of platelets in blood volume) will also be calculated, and (3) *Arterial O*_2 _*Saturation *will be assessed at rest and continuously during exercise using pulse oximetry (Biox 3700, Ohmeda Medical, Boulder, CO), which provides the most accurate noninvasive assessment of blood arterial O_2 _saturation levels. Pulse oximetry works on the principle of the red and infrared light absorption characteristics of oxygenated and deoxygenated Hb. Normal range is 96% to 100% at sea level for healthy humans.

**Skeletal Muscle Function **will be determined by: (1) *Fiber Type Distribution *will be determined by tissue biopsy of the vastumus laterialis using a modified Bergstrom needle technique [48]. Biopsy sites will be first anesthetized with a 2% lidocaine solution. Next, a 0.5 cm incision will be made through the skin and fascia lata. All samples will be prepared immediately by weighing and then dividing the samples for subsequent analysis as previously described[48]. A portion will be mounted in cross-section in optimal temperature compound (OCT) media immediately prior to being frozen in isopentane cooled by liquid nitrogen for fiber type determination. Specifically, myosin heavy-chain (MHC) isoforms I, IIa, and IIx will be identified by order of migration as described by Duscha et al [48]. Gels will be scanned electronically, and relative proportions of MHC isoform will be measured using NIH image 1.60 for Macintosh and Jandel PeakFit for Windows, (2) *Oxidative Capacity *(*Enzymology*) will be assessed via maximal activities of several oxidative pathways representative of different energy pathways using frozen tissue samples as previously described [48]. Phosphofructokinase and succinic dehydrogenase activities will be performed on fresh homogenates while the enzymes malate dehydrogenase and 3-hydoxyl-Co-A dehydrogenase will be performed on the frozen homogenates stored at -80°C, and (3) *Lower Extremity Maximal Muscular Strength *will be assessed as a voluntary one-repetition maximum (1-RM) using the following exercises: (1) leg press, (2) leg extension, and (3) leg curl. These tests will be repeated twice and the heaviest weight lifted while adhering to strict technique and form will be used as the score.

### Tracking and Monitoring of Adverse Events

Tracking and monitoring of adverse events will be assessed using the following methods: (1) during intervention sessions, all patients will receive one-on-one supervision and all adverse events (e.g., knee pain, back pain) will be recorded on the patient case report form (CRF). In addition, heart rate, blood pressure, and O_2 _saturation will be recorded prior to, during, and following every intervention session, (2) at the beginning of each week, the exercise physiologist will spend the first 10 minutes of every session discussing any potential negative side-effects of the intervention assignment and any injuries that may have occurred. All events will be recorded in the patient CRF, (3) every six months a meeting of all investigators will be scheduled to review and discuss all reported non-serious and serious adverse events for early identification of negative issues and development of solutions. All serious adverse events will be immediately reported to Duke IRB and immediately circulated to all study investigators for appropriate discussion, and (4) early stopping rules in response to a differential higher frequency of adverse events in a particular study group.

### Statistical Considerations

#### Sample Size Calculation

This randomized phase II trial will accrue 160 subjects with postsurgical NSCLC over an accrual period of ~48 months. For each of the primary and secondary endpoints, three separate t-tests will be used to compare each experimental arm to the control arm in mean change across time of the endpoint. For each endpoint, the overall alpha level will be controlled at a two-sided 0.05 by using Holm's procedure[49]. That is, Holm's procedure first ranks the three p-values from lowest to highest. The first (lowest) p-value has to be less than 0.05/3 (0.0167) to be significant and permit continuation to the other t-tests. The Holm's procedure continues sequentially in this fashion using alpha levels of 0.05/2 (0.025) and 0.05/1 (0.05) for the remaining two t-tests, respectively. Power for this study is defined as the probability that at least one of the three t-tests of the arm effect on VO_2peak _is significant; in other words, power is the probability that the first of the 3 ordered t-tests are significant. We assume that change in VO_2peak _will have a standard deviation of 3.0 mL.kg.^-1^min^-1 ^as observed in our pilot work. Statistical power depends upon the configuration of mean change in VO_2peak _across the 4 arms. Thus, for example, 80% power is obtained when the mean change in VO_2peak _across Arms 1, 2, 3, and 4 is 0.60, 0.60, 2.10, and 0.0 (mL.kg.^-1^min^-1^), respectively.

#### Analytic Plan

The principal analysis of the study endpoints will employ the intention-to-treat (ITT) approach. The ITT analysis will include all randomized participants in their randomly assigned allocation. The intervention group assignment will not be altered based on the participant's adherence to the randomly allocated study arm. Patients who are lost-to-follow-up will be included in all primary and secondary analyses by assuming zero change across time. For the primary analysis, a multiple regression model will be used to regress change in VO_2peak _on study group, the baseline value of the endpoint, and other pertinent baseline variables that may influence change in the study endpoints (e.g., co-morbid conditions/medications, self-reported exercise history, age).

## Discussion

### Methodological Considerations

Several issues were considered when designing LUNGEVITY. First, was the decision to investigate exercise in patients with operable (early) or inoperable (advanced) NSCLC. The majority of patients (~75%) diagnosed with NSCLC present with inoperable (advanced) disease. From a population health perspective, exercise trials in inoperable disease are likely to greater impact in comparison to those in patients with operable disease. Our decision to target early-stage patients was based on several important factors, primarily, exercise training safety and patient eligibility (recruitment). Inoperable NSCLC patients have poor prognosis and commonly present with significant smoking-related comorbid disease, advanced disease and symptoms, and are heavily treated with aggressive chemotherapy and concurrent radiotherapy [37]. As a result, the majority present with KPS scores <70% which may preclude participation in a moderate-intensity exercise training program and increase the risk of an exercise-related adverse [37]. Accordingly, it is prudent to focus on patients with operable disease; these patients have, in general, better performance status (and prognosis) and issues of NSCLC survivorship are becoming increasingly important aspect of multidisciplinary care. A second important consideration was the decision to investigate the efficacy of exercise following completion of, as opposed to, during adjuvant therapy. In our pilot work, aerobic training was associated with a 2% and 11% improvement in VO_2peak _among patients receiving or not receiving chemotherapy, respectively for postoperative NSCLC[17]. Clearly, without a non-intervention control group, it is not known whether maintenance of VO_2peak _during chemotherapy is important. Nevertheless, given the lack of benefit of exercise training during chemotherapy, subjects in LUNGEVITY will be recruited ≥6 months following the completion of adjuvant therapy, if appropriate.

### Ancillary Studies

A number of ancillary studies are planned for LUNGEVITY. First, as part of the informed consent process, a sample of white cells (buffy coat) will be collected from each subject in order to conduct genetic studies. A question of great interest that has not yet been addressed in the cancer populations is assessing genetic contributions to inter-individual differences in response to exercise training. Second, we will conduct ancillary study to examine predictors of adherence to the interventions as well as extent of exercise 'drop-in' in the attention control group across different exercise protocols in NSCLC. To address this question, we will adopt the guiding principles of the theory of planned behavior (TPB)[50]. This data will be used to inform the design and implementation of future trials. Third, in addition to assessing the physiological mediators of the exercise - VO_2peak _relationship, LUNGEVITY also provides a unique opportunity to examine which psychological variables that may mediate the exercise - PROs relationship. To this end, we will assess whether self-efficacy, affect, and social support mediate the effect of the exercise interventions and attention-control on anticipated improvements in PROs. Finally, we will include pilot studies to investigate the effects of exercise training on changes in peripheral gene expression (from analysis of whole blood mRNA) using high-density mRNA microarrays. Such studies will provide insight into the molecular mechanistic properties of exercise in NSCLC.

### Summary

The past decade has witnessed a dramatic increase in clinical and research interest of the application of exercise following a cancer diagnosis [51-58]. Recent systematic reviews conclude that exercise is a safe and feasible supportive intervention to improve symptom control and cardiorespiratory fitness in cancer patients with early-stage disease either during or following the completion of adjuvant therapy [59-63]. Moreover, recent observational studies provided the first evidence that regular exercise (i.e., 30 minutes of brisk walking at least 5 days/week) may be associated with substantial reductions in cancer-specific mortality and all-cause mortality among patients with early breast and colorectal cancer following the completion of adjuvant therapy [64-68]. Despite this growing evidence, investigation of the role of exercise training following a diagnosis of NSCLC remains limited.

The pathophysiology of NSCLC together with conventional therapeutic management is associated with unique and varying degrees of adverse physiological impairments that dramatically reduce patient's ability to tolerate exercise. Poor VO_2peak _likely predisposes to increased susceptibility to other common age-related diseases, greater symptoms, poor QOL, and even premature death. In recent years, a limited number of pilot studies have emerged that provide 'proof-of-principle' that supervised aerobic training is a safe and feasible supportive intervention associated with improvements in cardiopulmonary function and select patient-reported outcomes in postsurgical NSCLC. In addition, VO_2peak _may be a strong, independent predictor of long-term all-cause mortality in this population. Against this background, LUNGEVITY was designed to identify the most effective type of exercise training to improve VO_2peak _and to elucidate the physiologic mechanisms underlying this improvement. To our knowledge, LUNGEVITY will be the first trial to compare different types of exercise training protocols in NSCLC as well as the first to study the effects of exercise on changes in the organ components that govern exercise tolerance in any cancer population. In totality, LUNGEVITY will address many critical unanswered questions regarding the role and mechanistic properties of exercise in NSCLC and will set the stage for more definite trials. In the long-term, we hope that this research will contribute to the establishment of clinical exercise therapy rehabilitation guidelines for patients across the entire NSCLC continuum.

## Competing interests

The authors declare that they have no competing interests.

## Authors' contributions

LWJ: conception and design, drafting of manuscript, and final approval for publication. NDE: conception and design and final approval for publication. WEK: conception and design and final approval for publication. AP: conception and design and final approval for publication. JC: conception and design and final approval for publication. JAB: conception and design and final approval for publication. BLP: drafting of manuscript and final approval for publication. PSD: conception and design, drafting of manuscript, and final approval for publication.

## Pre-publication history

The pre-publication history for this paper can be accessed here:

http://www.biomedcentral.com/1471-2407/10/155/prepub
